# Comprehensive two-dimensional gas chromatography with flow modulator coupled via tube plasma ionization to an atmospheric pressure high-resolution mass spectrometer for the analysis of vermouth volatile profile

**DOI:** 10.1007/s00216-023-04688-6

**Published:** 2023-04-15

**Authors:** Juan F. Ayala-Cabrera, Lidia Montero, Taher Sahlabji, Oliver J. Schmitz

**Affiliations:** 1 Applied Analytical Chemistry, University of Duisburg-Essen, Universitatsstr. 5, D-45141 Essen, Germany; 2 Teaching and Research Center for Separation, University of Duisburg-Essen, Universitatsstr. 5, 45141 Essen, Germany; 3 Department of Chemistry, College of Science, King Khalid University, P.O. Box 9004, Abha, 61413 Saudi Arabia

**Keywords:** Aroma profile, Vermouth, Tube plasma ionization, Flow modulator, GC × GC

## Abstract

The analysis of complex samples is a big analytical challenge due to the vast number of compounds present in these samples as well as the influence matrix components could cause in the methodology. In this way, comprehensive two-dimensional gas chromatography coupled to mass spectrometry (GC × GC–MS) is a very powerful tool to achieve the characterization of complex samples. Nevertheless, due to possible coelutions occurring in these matrices, mixed spectra are generally obtained with electron ionization (EI) which could extremely complicate the identification of the analytes. Thereby, new methodology setups are required to improve the confidence on the identification in non-targeted determinations. Here, we present a high-throughput methodology consisting of GC × GC with flow modulation coupled to high-resolution atmospheric pressure mass spectrometry (HRMS) via a novel tube plasma ion source (TPI). The flow modulator allows to easily automate the GC × GC method compared to traditional cryo-modulators, while the soft ionization provided by TPI helps to preserve the [M]^+•^ or [M+H]^+^ ions, thus increasing the confidence in the identification. Additionally, the combination of a flow modulation with an atmospheric pressure mass spectrometer significantly improves the sensitivity over flow modulated GC × GC-EI-MS methods because no split is required. This methodology was applied to the analysis of a complex sample such as vermouth where the volatile profile is usually considered by consumers as a product quality indicator since it raises the first sensations produced during its consumption. Using this approach, different classes of compounds were tentatively identified in the sample, including monoterpenes, terpenoids, sesquiterpenoids and carboxylic acid, and carboxylate esters among others, showing the great potential of a GC × GC-TPI-qTOF-MS platform for improving the confidence of the identifications in non-targeted applications.

**Supplementary Information:**

The online version contains supplementary material available at 10.1007/s00216-023-04688-6.

## Introduction

The high complexity of some matrices such as food, environmental, or biological samples could complicate their analytical determination. From an instrumental and data treatment point of view, chromatographic coelutions lead to mixed mass spectra that might difficult the identification of the compounds, especially when hard ionization techniques such as electron ionization (EI) are used. Moreover, depending on the ion source selected, ion suppression effects could become critical, thus hindering the detection of analytes. Thereby, powerful analytical techniques that ensure both a high chromatographic resolution and a selective and sensitive determination are required when dealing with the characterization of complex samples. In this way, comprehensive two-dimensional gas chromatography (GC × GC) coupled to mass spectrometry has become a powerful technique to enhance the targeted analysis of complex samples or to identify differences and quality biomarkers between different samples [1–6]. In GC × GC analysis, the transferring process from the first dimension (^1^D) to the second dimension (^2^D) should be fast and reliable. Among the modulators used, valve-based modulators are gaining more relevance since they simplify the methodology, helping on the automatization since cryogenic solvents (i.e., liquid N_2_) are no longer required and reducing the costs compared with the most common thermal modulators (cryogen-based longitudinally modulated cryogenic system and the 2- and 4-jet modulators) [7, 8]. These valve-based modulators are generally classified in forward (FFF) and reverse flow fill/flush (RFF) modulators. FFF modulators elute the ^1^D effluent accumulated in the collection channel in the same direction than the loading step, while, in RFF modulators, the ^1^D effluent is eluted in the opposite direction, thus helping the focusing of the analytes in the ^2^D column especially when analyzing samples containing a wide dynamic concentration range [9]. Although these flow modulators are able to provide a good peak capacity and sensitivity, one of the main issues regards with their compatibility with mass spectrometry (MS) due to the high ^2^D flow rates (20 mL min^−1^). Thus, flow splitting before the MS entrance using vacuum ionization techniques such as EI is generally required, reducing the sensitivity of the method [10].

Traditionally, GC has been coupled to MS by vacuum ionization techniques like EI, allowing the identification of the ionized compounds by MS libraries (i.e., NIST). However, as mentioned above, these hard ionization sources produce a heavy fragmentation that may hamper the identification of the analytes in complex sample matrices. In this sense, atmospheric pressure ionization (API) sources can offer great potential in GC-MS determinations since these soft ionization techniques usually preserve the molecular or quasi-molecular ion. Moreover, their flexibility for the coupling of GC with advanced high-resolution mass spectrometry (HRMS) systems, usually limited to liquid chromatography applications [11, 12], enhances the capabilities of these sources for non-targeted applications. Among API sources, plasma-based sources are gaining more attention because they have shown a great ionization efficiency for a wider range of compounds than other API techniques [11]. Although plasma-based sources have been widely used for ambient ionization mass spectrometry [13], their application using GC-HRMS is still quite limited. Recently, our research group has developed a tube plasma ionization (TPI) source for GC-qTOF-MS coupling which allows the determination of a wide range of compounds [14]. Moreover, the operation of this source at atmospheric pressure conditions could help to improve the performance of flow modulators for GC × GC analysis since the ^2^D flowrate is negligible compared with the high flows required in the source, thus avoiding the split of the eluate before the MS determination. Thereby, this analytical platform could be a good strategy to simplify the analysis of complex samples.

In this work, the characterization of the aroma profile of commercial vermouth has been comprehensively evaluated by GC × GC-qTOF-MS using an RFF modulator. Due to the complex volatile profile of vermouth, advanced extraction and analytical tools are required. Thus, both the extraction process and GC × GC separation, including flow modulator parameters, have been carefully optimized. Additionally, the GC × GC-HRMS coupling has been done by using the novel TPI source to achieve a selective and wide-scope ionization and obtain the aroma profile identification of vermouth.

## Materials and methods

### Reagents and standards

Dichloromethane for HPLC grade (99.8%) was purchased from Thermo Fisher Scientific (Dreieich, Germany), while MilliQ water was obtained from a Sartorius ultrapure water system (Goettingen, Germany) (resistivity 18.2 M Ω cm^−1^). 1,4-Dichlorobenzene (≥ 99.9%), used as recovery standard, and Supelco 37-component FAMES mix (200–600 mg L^−1^), used as internal standard, were acquired from Merck (Darmstadt, Germany). The working solutions were prepared by adequate dilution of the recovery and internal standards in dichloromethane and stored at  − 20 °C before the analyses. Sodium chloride for analysis (100%) was obtained from Bernd Kraft (Duisburg, Germany). Moreover, helium Alphagaz 1 (≥ 99.999%), used as the carrier gas, argon Arcal Prime Smartop (≥ 99.999%), employed as the discharge gas, and nitrogen Alphagaz 1 (≥ 99.999%), used as the auxiliary gas in the TPI source, were supplied by Air Liquide (Oberhausen, Germany).

### Sample and sample preparation

Vermouth (18% *v/v* ethanol) was bought in a local store in Essen (Germany) in 2022. The sample was stored at  − 20 °C before extraction and analysis to avoid any loss of the most volatile analytes. A headspace solid-phase microextraction (HS-SPME) was considered for the recovery of VOCs from the vermouth. The HS-SPME was carried out following the procedure described by Zhu et al. [15] with slight modifications. Briefly, 2 mL of vermouth were diluted with 8 mL of MilliQ. Then, the sample was spiked with 20 µL of an appropriate concentration of the internal standards (1,4-dichlorobenzene and FAMES mix) to reach a concentration in the final extract of 5 µg L^−1^ and 4–12 µg L^−1^, respectively. Then, 2.5 g of sodium chloride were added, and the sample was equilibrated for 30 min at 45 °C. Then, the sample was extracted using a divinylbenzene/carboxen/polydimethylsiloxane (DVB/CAR/PDMS) fiber (1 cm, 50/30 μm; Supelco, Bellefonte, PA, USA) for 30 min at the same temperature with constant stirring. Finally, the fiber was injected into the GC injector for thermal desorption for 3 min, and it was left in the injector for another 30 min.

### Instrumentation

The analyses of the vermouth were conducted on an 8890 GC gas chromatograph, equipped with an 7693A ALS autosampler and a reversed flow modulator (Agilent Technologies, Santa Clara, CA, USA). The GC was coupled to a flame ionization detector (FID, Agilent Technologies) for both sample extraction and chromatographic separation optimizations. Additionally, for the identification of the aroma profile, the gas chromatograph was coupled to a 6546 LC/Q-TOF high-resolution mass spectrometer (Agilent Technologies) interfaced with the homemade TPI source described elsewhere [14]. Briefly, the TPI source is based on an inverse low-temperature plasma configuration. A stainless-steel needle electrode is positioned inside a quartz tube, also known as dielectric, where the argon discharge gas is passing through. When fast changes of high voltage are applied to the pin electrode, the dielectric is polarized, and the gas ignites by means of a dielectric barrier discharge (DBD). The GC × GC separation was carried out using a DB-5MS (30 m × 0.25 mm ID; 0.25 µm, Agilent Technologies) as ^1^D column, SLB-IL60 (5 m × 0.25 mm ID; 0.20 µm, Merck Supelco, Darmstadt, Germany), as ^2^D column, and a deactivated silica capillary (4.2 m × 0.1 mm ID, Agilent Technologies) as restrictor. The He carrier gas flow rates were set at 0.5 mL min^−1^ for the ^1^D and 20 mL min^−1^ for the ^2^D, respectively. The temperature program was fixed as follows: from 40 °C with 5 °C min^−1^ to 300 °C (hold for 10 min) (run time: 62 min). The injector temperature was set at 250 °C (splitless, 3 min). The modulation period and the injection time were set at 2.5 s and 0.12 s, respectively. Moreover, the transfer line temperature was fixed at 280 °C.

TPI was operated using argon as discharge gas at 200 mL min^−1^. The plasma working conditions consisted of a voltage of 2.5 kV, a frequency of 12.5 kHz, and a pulse width of 2 µs. Moreover, an N_2_ auxiliary gas at 400 mL min^−1^ was used to guide the ions through the plasma region and toward the MS inlet [16]. The dry gas flow rate and temperature were set at 13 L min^−1^ and 250 °C to avoid any condensation issues in the source, while the spray shield was not used for GC × GC-HRMS analyses. The fragmentor, skimmer, and octapole 1 RF Vpp voltages were set at 125, 65, and 750 V, respectively. The MS acquisition was done in full-scan data-dependent acquisition in both positive and negative ion mode. Both full-scan and MS/MS acquisitions were performed from 20 to 750 m*/z* using an acquisition rate 20 and 15 spectra s^−1^, respectively. Regarding the MS/MS acquisition, a maximum of 2 precursors were chosen per cycle (precursor threshold: 10,000 counts), and no isotope model was applied to allow the potential fragmentation of both [M]^+•^ and [M+H]^+^ ions. The collision energy was set according to a linear curve in a mass-dependent manner ranging from 11 eV at *m/z* 20 to 47.5 eV at *m/z* 750. The qTOF was calibrated daily to ensure mass accuracy with the ESI-L Low concentration tunning mix solution (1:10 *v/v*) using the Agilent Dual Jet Stream electrospray source from Agilent Technologies. The GC × GC was controlled using the OpenLab software, while the qTOF control, data acquisition, and processing were done using the Agilent MassHunter Workstation 10.0 software.

## Results and discussion

Vermouth consists of the maceration of dried herbs, barks, seeds, and leaves from aromatic and bitter herbs in white wine and neutral spirits for several weeks. This ethanolic maceration gives rise to the extraction of volatile and polar compounds present in the herbs that increase the already rich wine chemical profile in relevant bioactive compounds [17, 18]. Although wine is well characterized, there is very few information about the chemical composition of vermouths, and more studies are required to evaluate the properties of these beverages [19]. Indeed, the volatile profile of wine-related beverages defines the quality and the sensations produced during its consumption [20]. Thus, here we propose the use of GC × GC-TPI-qTOF analytical platform to help on the elucidation of the volatile profile of a vermouth sample.

### Method set-up optimization

**Fig. 1:**
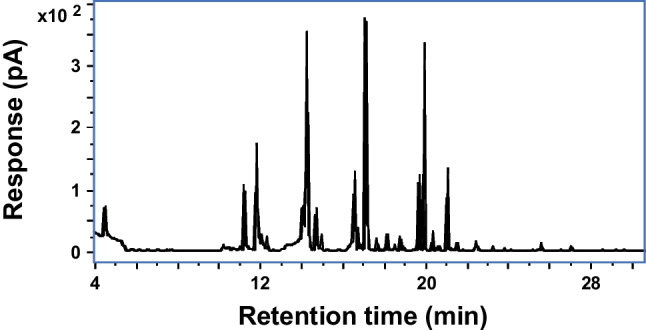
^1^D GC-FID chromatogram of vermouth using a DB-5MS (30 m × 0.25 mm ID; 0.25 µm) after HS-SPME (DVB/CAR/PDMS) extraction

The extraction of volatile organic compounds (VOCs) is a critical and challenging step considering the large variety of different volatile molecules that form wine and vermouth profiles as well as the low concentrations in which most of these compounds are usually found. Liquid-liquid extraction (LLE) and solid-phase extraction (SPE) have commonly been used for the recovery of the VOCs from wine-related matrices [21]. However, in recent years, the use of headspace solid-phase microextraction (HS-SPME) has been increasing since it is cheaper, faster, it requires less sample manipulation than LLE and SPE, and it does not use organic solvents, which follows the principles of green chemistry [22]. Among the fibers used, DVB/CAR/PDMS fiber which contains three adsorbents is generally selected, extending the range of VOCs extracted from food items [21, 23, 24]. In this work, the extraction of VOCs from the vermouth was adapted from Zhu et al. [15] as mentioned above. To ensure a constant recovery of the analytes from vermouth, the recovery yield determined as the ratio between the response of internal standard spiked at 1 mg L^−1^ in a blank and a vermouth sample was established as a quality control (*n* = 3). 1,4-Dichlorobenzene was selected since dichlorobenzenes has been widely used as internal standard for aroma analysis [15, 25, 26]. Under these conditions, the recovery was 98 ± 4% which ensures the high quality of the results. Additionally, as can be seen in the ^1^D chromatogram obtained by GC-FID (Fig. 1), the VOC profile of the vermouth is very complex, thus requiring the use of more powerful separation techniques such as GC × GC.

For the GC × GC separation, a non-polar DB-5MS (poly(5% phenyl/95% dimethyl arylene siloxane), 30 m × 0.25 mm ID; 0.25 µm) was selected in the ^1^D, while a highly polar ionic liquid (IL) phase consisting of the SLB-IL60 (1,12-di(tripropylphosphonium)dodecane bis(trifluoromethylsulfonyl)imide; 5 m × 0.25 mm ID; 0.2 µm) was chosen as ^2^D column. The use of an ionic liquid (IL) stationary phase in the ^2^D provides a low separation correlation with the selected ^1^D; at the same time, it has a higher thermal stability (up to 300 °C) compared with other polar stationary phases such as polyethylene glycol-based columns (up to 260/270 °C) [27, 28]. The separation of the VOC profile was achieved using common ^1^D (0.5 mL min^−1^) and ^2^D (20 mL min^−1^) He flow rates for reverse fill/flush modulators, whereas the temperature program was set as follows: 40 to 300 °C (held 10 min) at 5 °C min^−1^. One of the critical points when working with valve-based modulators is the optimization of the modulation parameters.

Regarding the injection time, 0.12 s were enough to achieve good and narrow modulated peak shapes, whereas to maximize the separation and avoid wrap-around effects, two modulation times (2.5 and 3 s) were considered. The evaluation of the effect of these two modulation periods was carried out by comparing the practical peak capacity (^2D^n_c_,_practical_) [29]. As can be seen in Table 1, the ^2D^n_c,practical_ calculated for the analysis done using 2.5 s modulation time was 1.7 times higher (^2D^n_c,practical_ = 7006) than the one using 3 s (^2D^n_c,corrected_ = 4140). This fact is related to the peak capacity reduction produced when higher modulation times are used. In this case, an undersampling effect occurs, and the obtained separation of peaks in the ^1^D can be lost during the modulation process giving rise to broader non-separated peaks. As can be seen in Table 1, while the peak capacity of the ^2^D (^2^n_c_) for both modulation times was very similar (24 and 20 for the analysis carried out at 2.5 and 3 s modulation time, respectively), the peak capacity of the ^1^D (^1^n_c_) was much lower when a higher modulation period was used (^1^n_c_ = 231) in comparison with the shorter modulation time (^1^n_c_ = 374). Thereby, the modulation time was set at 2.5 s. Thus, the GC × GC setup optimized in this work provided very high peak capacity values and high orthogonality for the separation of the vermouth aromatic profile.

**Table 1:** Peak capacity achieved using both modulation times

Separation parameter	Modulation time (s)^a^	
	2.5	3.0
^1^*n*_c_	374	231
^2^*n*_c_	24	20
^2D^*n*_c_,_practical_	7006	4140

^a^Injection time: 0.12 s

### GC × GC-HRMS coupling via TPI for the analysis of vermouth

One of the main gaps of flow modulators has been their coupling with MS systems. The high flow rates required in the ^2^D (ca. 15–30 mL min^−1^) are not compatible with those MS instruments working with vacuum ionization techniques such as electron ionization [30]. To sort out this problem, Tranchida et al. [10] proposed the use of a longer external accumulation loop for collecting the ^1^D eluate. This homemade flow modulator leads to a reduction of the ^2^D flow rate up to 6–8 mL min^−1^ and an improvement of the peak shapes. However, when using current available flow modulators, a split of the flow is required before the connection of the GC × GC with the mass spectrometer, leading to a loss of sensitivity of the whole setup. In contrast, when working with API techniques such as TPI, this ^2^D flow rate is negligible compared with other gases used in the source such as the N_2_ auxiliary gas and dry gas (ca. 0.2–13 L min^−1^) [31]. Therefore, the split is no longer required, which allows to detect all the eluate coming from the GC, and consequently, it does not reduce the sensitivity of the method.

**Fig. 2:**
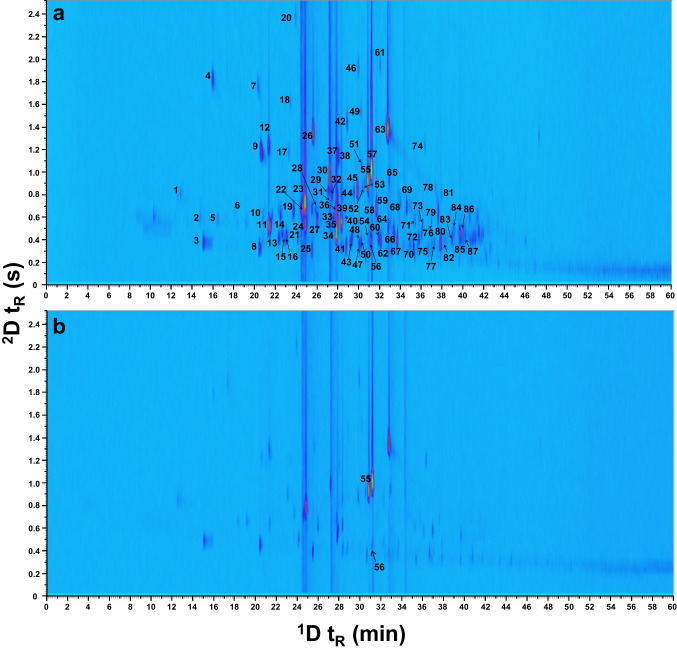
GC × GC-HRMS chromatogram of vermouth (1:4 *v/v* diluted with H_2_O) using a DB-5MS (30 m × 0.25 mm ID, 0.25 µm) as ^1^D column and SLB-IL60 (5 m × 0.25 mm ID, 0.20 µm) as ^2^D column (modulation period: 2.5 s; injection time: 0.12 s) in **a** positive and **b** negative TPI mode

Under these setup conditions, Fig. 2 shows the GC × GC-HRMS chromatograms using the TPI source in positive and negative ion modes. In these results, it can be observed that most of the compounds were only ionized in positive TPI mode, which shows a more complex profile. However, although in this case the positive mode provided better ionization efficiency, the possibility to switch the polarity in API sources provides additional information and advantages to carrying out the identification of VOCs, especially those compounds that are ionized in both ionization modes.

Regarding the separation and the peak shape, it was observed that some of the peaks showed a significant peak tailing in the ^2^D. This effect could be related to the high concentration of some of the compounds present in the sample that are transferred to the ^2^D short column producing an overloading of the column. At this point, different dilutions of the sample were injected using the optimized method as can be seen in the GC × GC chromatograms of the vermouth sample with different dilution factors (see electronic supplementary material (ESM), Fig. S1). The higher the dilution was, the less peak tailing was observed in the chromatogram.

**Fig. 3:**
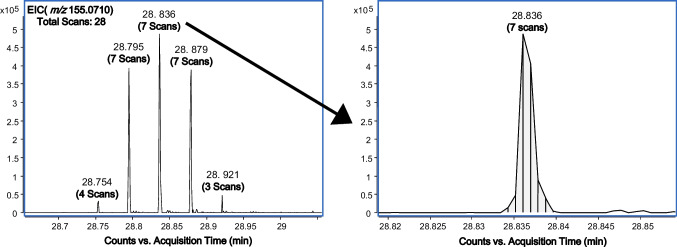
Peak shape of an average modulated peak (*m/z* 155.0710) achieved by GC × GC-TPI-HRMS (qTOF)

However, injecting the sample in a low concentration produced a loss of sensitivity of the less concentrated compounds due to the large range of metabolite concentrations present in the sample. Figure S2 shows the loss of sensitivity of one of the low concentrated compounds after the 1:100 dilution. This peak presents a good peak shape tailing and adequate S/N intensity when the concentrated sample is injected. However, if a dilution is done in the sample, the signal is close to the noise with the risk of losing the information of this compound during the filtering of the data treatment. Therefore, the analysis of the sample diluted in a 1:4 *v/v* ratio with water was used for the chemical identification of the VOCs in vermouth. In addition, modulated peaks showed very narrow peaks widths (ca. 0.36 s) which require very fast mass analyzers to ensure a good quality of the acquired data. Generally, it is assumed that a well-defined ^1^D GC peak may have at least 8–10 scans for quantification purposes, although this number could be slightly lower for qualitative analysis. To fulfill this criterion, the scan rate was set at 20 spectra s^−1^ for the full-scan acquisition. As can be seen in Fig. 3 for an average intense peak eluted at the middle of the temperature program, there are around 7 scans per modulated peak. Considering that the peaks are modulated at least 3–4 times, around 20–30 spectra per ^1^D peak can be expected, which is high enough to carry out the identification of VOCs in vermouth. Additionally, the outstanding detection capability that TPI has shown for a wide range of analytes [14] may ensure the detection of flavoring compounds even at low concentration ranges. The repeatability of the HS-SPME GC × GC-TPI-qTOF-MS methodology has been evaluated by measuring the area of the internal standard (1,4-dichlorobenzene) in the sample (*n* = 3). The relative standard deviation (RSD) was 8.2% which shows the good performance of the determination.

Thus, this analytical platform provides a clear separation and selective and sensitive detection of the VOC profile of vermouth in 60 min, which can be very useful not only for characterization purposes but also to develop high-throughput authentication approaches.

### Identification of VOCs by GC × GC-TPI-MS/HRMS

**Fig. 4:**
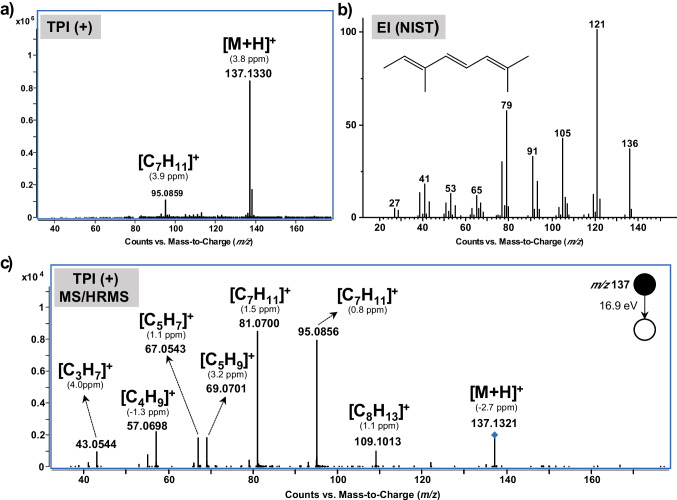
Mass spectra of (4E,6E)-allocimene obtained by **a** TPI ( +) in full scan, **b** EI (*obtained from the NIST library*), and **c** MS/HRMS experiments after TPI ( +)

The developed methodology was applied to the VOC profile characterization of the vermouth sample. The diluted sample (1:4 *v/v*) was extracted by HS-SPME (DVB/CAR/PDMS) and analyzed by GC × GC-qTOF-MS (*n* = 3) in both, positive and negative TPI modes. The confidence of the identifications was established according to the criteria reported by Schymanski et al. [32]. Although structure assignments based on the monoisotopic mass, the isotopic cluster match, and the ring double bond equivalent (RDBE) enhance the confidence level, the assignment of probable structures (level 2) requires literature or MS library match. However, this is one of the main gaps of GC-API-MS methodologies since the number of MS libraries available is quite low [11, 33], or in the case of new API sources such as TPI, they do not even exist. For that reason, the establishment of tentative candidates (level 3) was achieved by the comparison of experimental MS/HRMS spectra with those predicted by in silico methods [33, 34] using MetFrag [35]. To propose one structure as a tentative candidate, a minimum MS/HRMS match score of 80% was considered. Moreover, to ensure the correct assignment, the mass accuracy was fixed at 5 ppm for precursor molecules, while for product ions, it was set at 5 and 10 ppm for *m/z* higher and lower than 100 Da, respectively. In addition, the linear retention indexes (LRI) were determined and compared with those proposed in the literature and mass spectral libraries (i.e., NIST) by means of the absolute difference (ΔLRI_theo_) to increase the confidence in the identification. The proposed annotations were also filtered considering their relevance for food and aroma analysis using the FoodDB database [36]. Following all these conditions, compounds tentatively identified in the vermouth sample, corresponding with the peaks clearly identified in the 2D plot, are shown in Table 2. The most abundant classes of compounds identified were monoterpenes, terpenoids, and sesquiterpenoids, carboxylic acids and carboxylate esters, alkylbenzenes, phenols and derivatives, and aromatic aldehydes. Most of these compounds are flavor agents which might be strongly related to the organoleptic properties of the vermouth. All the compounds detected in positive mode presented a very clear [M+H]^+^ ion as the base peak of the mass spectra, as shown in Fig. 4 (compound identified as (4E,6E)-allocimene), except for the compound piperonal, that showed abundant [M+H]^+^ and the [M]^+•^. As can be observed, the use of TPI as ion source significantly decreased the in-source fragmentation, thus improving the selectivity of the methodology compared with those approaches based on the EI source (Fig. 4b). Moreover, the possibility to carry out tandem mass spectrometry experiments (MS/HRMS) using the GC × GC-TPI-qTOF-MS approach provided structural information that helps to increase the confidence on the annotation (i.e., (4E,6E)-allocimene MetFrag score: 84.3%). Although most of the analytes were only identified in positive TPI mode by means of the protonated molecule, some compounds could also be determined in negative ion mode. For instance, carvacrol led to the deprotonated molecule while Edulan I showed the [M-CH_3_]^‒^ ion as the base peak of the mass spectra. All this information, together with the well-defined retention times in both dimensions and the LRI, may increase the confidence level in the identification of these compounds.

GC × GC-TPI-qTOF-MS significantly increases both the sensitivity and the selectivity of the detection of aroma compounds. However, especially for some minor components of vermouth, the intensity could be still too low to acquire a good quality MS/HRMS spectrum. Even though TPI generally preserves the molecular/quasi-molecular ion, in some cases, in-source collision-induced dissociation (CID) fragmentation could be achieved (Fig. S3), depending on the lability of the compound [14]. When this happens and no or bad quality MS/HRMS spectrum was acquired, this fragmentation can be used to carry out in silico fragmentation comparison.

**Table 2:** VOCs tentatively identified in the vermouth sample

Peak ID	^1^D t_R_ (min)	^2^D t_R_ (s)	t_R_ total (min)	Molecular formula	*m/z*	Ion	Error (ppm)	RDBE^a^	MS/HRMS match (%)	LRI_exp_ (ΔLRI_theo_)	Candidate	Family of compound
1	12.866	0.839	12.880	‒	177.1856	‒	‒	‒	n.a.^b^	749 (‒)	n.i	‒
2	14.658	0.682	14.669	C_6_H_12_O_2_	117.0916	[M+H]^+^	5.0	0.5	100	826 (+ 3)	Methyl pentanoate	Ester
3	14.991	0.420	14.988	‒	223.0645	‒	‒	‒	n.a.^b^	846 (‒)	n.i	‒
4	15.908	1.836	15.939	C_5_H_4_O_2_	97.0289	[M+H]^+^	5.0	3.5	100	898 (+ 7)	Furfural	Aldehyde
5	16.366	0.734	16.378	C_7_H_14_O_2_	131.1072	[M+H]^+^	2.6	0.5	100	908 (+ 3)	Pentyl acetate	Ester
6	19.075	0.787	19.088	‒	225.0434	‒	‒	‒	n.a.^b^	953 (‒)	n.i	‒
7	20.241	1.731	20.269	C_6_H_6_O_2_	111.0445	[M+H]^+^	4.0	3.5	100	972 (+ 7)	5-Methylfurfural	Aldehyde
8	20.366	0.367	20.372	‒	297.0832	‒	‒	‒	n.a.^b^	974 (‒)	n.i	‒
9	20.533	1.206	20.553	C_7_H_7_O	107.0494	[M+H]^+^	2.4	4.5	100	976 (+ 10)	Benzaldehyde	Aldehyde
10	20.700	0.892	20.715	C_8_H_14_	111.1169	[M+H]^+^	0.7	1.5	n.a.^b^	980 (‒)	n.i	‒
11	21.283	0.629	21.293	C_8_H_16_O_2_	145.1226	[M+H]^+^	2.0	0.5	95	992 (− 6)	Ethyl hexanoate	Ester
12	21.283	1.259	21.304	C_7_H_10_O	111.0808	[M+H]^+^	3.2	2.5	n.a.^b^	992 (− 13)	2,4-Heptadienal	Aldehyde
13	22.241	0.472	22.249	C_10_H_16_	137.1330	[M+H]^+^	3.8	2.5	100	1001 (+ 7)	β-myrcene	Terpene
14	22.575	0.472	22.583	C_10_H_14_	135.1170	[M+H]^+^	3.5	3.5	91	1019 (− 3)	m-Cymene/ρ-Cymene	Alkylbenzene
15	22.616	0.472	22.623	C_10_H_14_	135.1173	[M+H]^+^	3.5	3.5	97	1020 (− 2)	m-Cymene/ρ-Cymene	Alkylbenzene
16	22.991	0.472	22.999	C_10_H_16_	137.1327	[M+H]^+^	1.6	2.5	85	1030 (0)	D-Limonene	Terpene
17	23.241	1.206	23.261	C_7_H_9_O_3_	141.0550	[M+H]^+^	2.7	3.5	100	1040 (− 5)	Ethyl furoate	Ester
18	23.366	1.521	23.391	C_7_H_12_O_3_	145.0860	[M+H]^+^	1.9	1.5	100	1044 (− 1)	Ethyl levulinate	Ester
19	23.658	0.734	23.670	C_8_H_12_O_2_	141.0916	[M+H]^+^	3.5	2.5	100	1052 (n.r.)	Cis-1,2-dihydro-3- cresol l	Ester
20	23.908	2.255	23.946	C_7_H_9_O	109.0650	[M+H]^+^	1.9	3.5	100	1061 (− 9)	o-/m-/ρ-Cresol	Phenol
21	24.075	0.367	24.081	‒	299.0620	‒	‒	‒	n.a.^b^	1065 (‒)	n.i	‒
22	24.533	0.734	24.545	C_8_H_12_O_2_	141.0911	[M+H]^+^	0.7	2.5	100	1079 (n.r.)	Ethyl-2-methyl-3,4-pentadienoate	Ester
23	24.783	0.787	24.796	C_8_H_12_O_2_	141.0913	[M+H]^+^	2.1	2.5	93	1094 (− 3)	Ethyl sorbate	Ester
24	24.825	0.577	24.834	C_10_H_12_O	149.0963	[M+H]^+^	1.4	4.5	100	1095 (n.r.)	Trans-Lachnophyllol	Alcohol
25	25.450	0.315	25.455	‒	371.1020	‒	‒	‒	n.a.^b^	1115 (‒)	n.i	‒
26	25.575	1.311	25.597	C_8_H_8_	105.0699	[M+H]^+^	0.2	4.5	100	1124 (‒)	Styrene isomers	Vinylbenzene
27	25.750	0.577	25.760	C_10_H_16_	137.1331	[M+H]^+^	4.5	2.5	90	1134 (+ 4)	(4E,6Z)-Allocimene	Terpene
28	25.950	0.629	25.960	C_10_H_16_	137.1328	[M+H]^+^	2.4	2.5	84	1147 (+ 3)	(4E,6E)-Allocimene	Terpene
29	26.991	0.892	27.006	C_10_H_16_O	153.1280	[M+H]^+^	4.0	2.5	80	1205 (+ 3)	Trans-dihydrocarvone	Ketone
30	27.158	0.996	27.175	C_8_H_14_O_4_	175.0971	[M+H]^+^	3.5	1.5	100	1209 (+ 3)	Fructone	Ketone
31	27.325	0.787	27.338	C_9_H_10_O_2_	151.0760	[M+H]^+^	4.3	4.5	95	1214 (− 2)	Methyl ρ-methylbenzoate	Benzoic acid
32	27.366	0.839	27.380	C_9_H_10_O	151.0761	[M+H]^+^	4.9	4.5	100	1215 (-8)	Ethyl benzoate	Benzoic acid
33	27.491	0.629	27.501	C_10_H_16_	137.1331	[M+H]^+^	4.5	2.5	n.a.^b^	1218 (‒)	n.i	‒
34	27.825	0.472	27.833	C_10_H_20_O_2_	173.1543	[M+H]^+^	4.0	0.5	90	1226 (+ 13)	Octyl acetate	Ester
35	27.825	0.524	27.833	C_10_H_20_O_2_	173.1536	[M+H]^+^	− 0.04	0.5	90	1226 (+ 1)	Methyl nonanoate	Ester
36	27.825	0.682	27.837	C_10_H_16_O	153.1278	[M+H]^+^	2.7	2.5	90	1226 (− 3)	Cis-Carveol	Terpene
37	27.950	1.154	27.969	C_9_H_10_O_2_	135.0808	[M+H]^+^	2.7	4.5	85	1230 (-4)	Chavicol	Phenol
38	27.991	1.101	28.009	C_9_H_10_O	135.0811	[M + H]^+^	4.9	4.5	95	1231 (− 13)	Cinnamyl alcohol	Alcohol
39	28.283	0.629	28.293	C_10_H_17_O_2_	169.1225	[M+H]^+^	1.1	2.5	81	1238 (− 6)	Ascaridole	Terpene
40	28.658	0.629	28.668	C_10_H_16_O	153.1276	[M+H]^+^	1.4	2.5	97	1248 (+ 8)	Neral	Terpene
41	28.741	0.367	28.747	C_10_H_16_	137.1329	[M+H]^+^	3.1	2.5	n.a.^b^	1250 (+ 6)	E-4-Decen-6-yne	Alkine
42	28.825	1.469	28.849	C_8_H_10_O_3_	155.0710	[M+H]^+^	4.7	3.5	n.a.^b^	1252 (− 3)	1-(2-furanyl)-3-butene-1,2-diol	Heterocyclic compound
43	29.241	0.420	29.248	C_11_H_14_O	163.1120	[M+H]^+^	1.6	4.5	n.a.^b^	1263 (+ 1)	2-Methyl-1-phenyl-1-butanone	Ketone
44	29.491	0.892	29.506	C_10_H_12_O_2_	165.0918	[M+H]^+^	4.8	4.5	92	1269 (+ 10)	Ethyl phenylacetate	Ester
45	29.783	0.892	29.798	C_10_H_14_O	151.1124	[M+H]^+^	4.3	3.5	100	1277 (+ 5)	Perillaldehyde	Terpene
46	29.908	1.941	29.940	C_8_H_14_O_5_	191.0912	[M+H]^+^	-1.0	1.5	100	1279 (+ 8)	Diethyl malate	Carboxylic acid
47	29.950	0.367	29.956	C_10_H_16_O	153.1276	[M+H]^+^	0.2	2.5	94	1281 (+ 9)	Geranial	Terpene
48	29.991	0.420	29.998	C_10_H_16_	137.1330	[M+H]^+^	3.8	2.5	80	1282 (n.r.)	3-Methylene-1,5,5-trimethylcyclohexene	Alkylbenzene
49	30.158	1.469	30.182	C_8_H_8_O_2_	137.0602	[M+H]+	3.6	4.5	100	1287 (nr)	3-(3-Furanyl)-2-methyl-2-propenal	Heterocyclic compound
50	30.200	0.367	30.206	C_10_H_16_	137.1331	[M+H]^+^	4.5	2.5	82	1287 (− 5)	1,3,5,5-Tetramethyl-1,3-cyclohexadiene	Alkylbenzene
51	30.200	1.101	30.218	C_10_H_16_O	153.1280	[M+H]^+^	4.0	2.5	97	1288 (− 8)	Perillyl alcohol	Terpene
52	30.241	0.944	30.257	C_9_H_16_O_4_	189.1124	[M+H]^+^	1.4	1.5	92	1289 (+ 6)	Diethyl glutarate	Ester
53	30.491	0.944	30.507	C_10_H_14_O	151.1121	[M+H]^+^	2.4	3.5	95	1292 (+ 1)	Thymol	Terpene
54	30.825	0.472	30.833	C_11_H_22_O_2_	187.1693	[M+H]^+^	0.2	0.5	100	1304 (+ 2)	Octyl propanoate	Ester
55	30.825	0.944	30.841	C_10_H_14_O	151.1115	[M+H]^+^	-1.6	3.5	94	1304 (+ 5)	Carvacrol	Terpene
					149.0979	[M‒H]^‒^	4.8	4.5	96			
56	31.116	0.472	31.124	C_13_H_20_O	193.1593	[M+H]^+^	3.1	3.5	93	1312 (− 2)	Edulan I	Benzopyrene
					177.1289	[M‒CH_3_]^‒^	2.3	4.5	n.a.^b^			
57	31.200	1.094	31.218	C_10_H_14_O	151.1118	[M+H]^+^	0.4	3.5	100	1315 (+ 1)	3-Caren-5-one	Terpene
58	31.574	0.678	31.585	C_8_H_9_O_3_	153.0546	[M+H]^+^	0.1	4.5	86	1325 (+ 6)	6-Hydroxy anisaldehyde	Arylaldehyde
59	31.616	0.682	31.627	C_8_H_8_O	187.1689	[M+H]^+^	-1.9	0.5	100	1326 (+ 1)	Methyl decanoate	Ester
60	31.825	0.472	31.833	C_13_H_20_O_2_	209.1540	[M+H]^+^	1.9	3.5	90^c^	1330 (− 13)	Cis-Chrysanthenyl propionate	Ester
61	31.991	1.888	32.022	C_9_H_16_O_2_	157.1229	[M+H]^+^	3.8	1.5	80	1331 (+ 7)	Nonalactone	Heterocyclic compound
62	32.033	0.420	32.040	C_12_H_18_O	179.1439	[M+H]^+^	4.8	3.5	100	1338 (− 5/n.r.)	Tricycloekasantal/3-Methyl-1-phenyl-3-pentanol	Alkylbenzene
63	32.741	1.364	32.763	C_8_H_6_O_3_	150.0311	[M]^+•^	-0.3	6.0	98	1340 (+ 10)	Piperonal	Aldehyde
					151.0394	[M+H]^+^	2.8	5.5	100			
64	32.783	0.524	32.792	C_10_H_16_	137.1330	[M+H]^+^	3.8	2.5	100	1350 (+ 12)	1,5,5-Trimethyl-6-methylene-cyclohexene	Alkylbenzene
65	32.825	0.892	32.840	C_10_H_12_O_2_	165.0915	[M+H]^+^	3.0	4.5	100	1361 (+ 4)	Eugenol	Phenol
66	33.450	0.472	33.458	C_12_H_22_O_2_	199.1700	[M+H]^+^	3.7	1.5	100	1364 (+ 8)	Citronellyl acetate	Alcohol
67	33.616	0.420	33.623	C_12_H_24_O_2_	201.1856	[M+H]^+^	3.4	0.5	100	1383 (− 1)	Hexyl hexanoate	Ester
68	33.908	0.734	33.920	C_11_H_14_O_2_	179.1068	[M+H]^+^	0.8	4.5	99^c^	1396 (− 3)	Methyl eugenol	Alkylbenzene
69	34.533	0.682	34.544	C_8_H_8_O_3_	153.0549	[M+H]^+^	1.8	4.5	100	1404 (0)	Vanillin	Phenol
70	35.200	0.367	35.206	C_15_H_26_O	223.2066	[M+H]^+^	4.2	2.5	97^c^	1440 (+ 8)	Ginsenol	Alcohol
71	35.283	0.524	35.292	C_13_H_22_O	195.1741	[M+H]^+^	1.2	2.5	90^c^	1451 (− 2)	Geranyl acetone	Terpene
72	35.700	0.420	35.707	C_15_H_26_O	223.2064	[M+H]^+^	4.0	2.5	95^c^	1479 (− 12)	Epicubebol	Sesquiterpene
					205.1959	[M+H-H_2_O]^+^	4.0	3.5	n.a.^b^			
73	35.991	0.472	35.999	C_14_H_20_O_2_	221.1540	[M+H]^+^	1.8	4.5	85/86	1485 (− 3/ + 5)	Dimethylbenzylcarbinyl butyrate/Thymol isobutyrate	Terpene
74	36.241	1.154	36.260	C_11_H_16_O_3_	197.1177	[M+H]^+^	2.4	3.5	100	1497 (n.r.)	Isobutyl 2-furanpropionate	Ester
75	36.575	0.315	36.580	‒	463.1311	‒	‒	‒	n.a.^b^	1508 (‒)	n.i	‒
76	36.908	0.524	36.917	C_14_H_21_O	206.1667	[M]^+•^	0.9	4.0	93	1518 (0)	α-Irone	Sesquiterpene
					207.1746	[M+H]^+^	1.2	3.5	92^c^			
77	37.200	0.367	37.206	C_13_H_26_O_2_	215.2015	[M+H]^+^	4.3	0.5	100^c^	1527 (+ 1)	Methyl dodecanoate	Ester
78	37.325	0.839	37.339	C_12_H_18_O	179.1434	[M+H]^+^	2.0	3.5	n.a.^b^	1531 (‒)	n.i	‒
79	37.533	0.682	37.544	C_11_H_12_O_3_	193.0866	[M+H]^+^	3.5	5.5	91^c^	1537 (n.r.)	Eugenyl formate	Phenol
80	37.825	0.420	37.832	C_15_H_24_O	221.1907	[M+H]^+^	3.2	3.5	100	1545 (+ 4)	α-Copaen-11-ol	Sesquiterpene
81	37.825	0.839	37.839	C_12_H_16_O_3_	209.1180	[M+H]^+^	3.7	4.5	100	1546 (+ 4)	Isoamyl salicylate	Ester
82	38.491	0.420	38.498	C_15_H_24_	205.1957	[M+H]^+^	3.0	3.5	100	1565 (+ 9)	Guaia-3,9-diene	Sesquiterpene
83	38.866	0.420	38.873	C_15_H_24_	205.1954	[M+H]^+^	1.6	3.5	80	1573 (+ 13)	Germacrene B	Sesquiterpene
84	39.075	0.524	39.084	C_15_H_24_O	221.1903	[M+H]^+^	4.1	3.5	93^c^	1583 (− 3)	β-Copaen-4α-ol	Sesquiterpene
85	39.616	0.472	39.624	C_15_H_24_	205.1960	[M+H]^+^	4.5	3.5	100	1590 (‒)	n.i	‒
86	39.991	0.524	40.000	C_15_H_22_O	219.1748	[M+H]^+^	0.4	4.5	94	1629 (− 3)	Turmerone	Sesquiterpene
87	40.158	0.420	40.165	C_15_H_22_	203.1801	[M+H]^+^	3.3	4.5	n.a.^b^	1637 (‒)	n.i	‒

^a^Ring double bond equivalent. ^b^Not acquired. ^c^Based on in-source CID fragmentation

In these cases, a minimum MS/HRMS match of 90% was required to ensure the correct annotation. Using this strategy, the confidence level on the identification of cis-chrysanthenyl propionate, methyl eugenol, ginsenol, geranyl acetone, epicubebol, α-irone, methyl dodecanoate, eugenyl formate, and β-Copaen-4α-ol could be increased without having a high-quality MS/HRMS acquisition.

The proposed aroma compounds have been related to different trends considering their main odor/tasting descriptors (see ESM, Table S1). On one hand, monoterpenes, terpenoids, and sesquiterpenoids are strongly related to floral and fruity aroma descriptors. Regarding aryl-aldehydes, those found in the vermouth sample (furfural, 5-methylfurfural, benzaldehyde, and heptadienal) are the main responsible for the almond odor. These compounds are present in natural sources like coffee, cacao, nuts, or some berries; however, furfural and methyl furfural can also be formed during the aging of the vermouth by Maillard and/or caramel reactions [37]. Alkylbenzenes and phenols showed different trends from sweet and citrus aroma compounds (i.e., styrene, 3-methyl-1-phenyl-3-pentanol, cymene, methyl eugenol, and vanillin), to spicy (i.e., eugenol and 4-vinylguaiacol), or even medicinal odor (i.e., chavicol and cresol). On the other hand, carboxylate esters are generally related to fruity aromas such as ethyl-2-methylpentanoate, so-called manzanate, diethyl malate, methyl pentanoate, pentyl acetate, ethyl furoate, ethyl levulinate, and ethyl sorbate, among others. Other compounds that may contribute to the fruity aroma are alcohol, esters (citronellyl aceate), and heterocyclic compounds like fructone, nonalactone, or piperonal. Thus, the different kinds of herbs, barks, seeds, and leaves used for the maceration will lead to a unique VOC profile of the vermouth analyzed. Therefore, the monitoring of these profiles using advanced analytical platforms such as GC × GC-TPI-qTOF-MS could help to identify potential biomarkers to differentiate between types of vermouths or even deal with potential authentication issues.

## Conclusions

The proposed GC × GC-TPI-qTOF-MS methodology has shown a great potential to the analysis of complex samples such as vermouth. The use of a reverse fill/flush modulator allows an easier automatization of the GC × GC separation at the same time; it keeps a good peak shape for the modulated peaks. The column combination consisted of DB-5MS and SLB-IL60 in first and second dimension, respectively. The use of this ionic liquid column in the ^2^D provided a high orthogonality at the same time; it helps to reach higher temperatures (up to 300 °C) compared to other polar stationary phases such as polyethylene glycol-based columns (ca. up to 260/270 °C). Moreover, the combination of these powerful separation technique with an API source such as TPI helps to overcome the loss of sensitivity observed in GC × GC-EI-MS systems using flow modulators since there is no need to split the flow prior to the analysis with atmospheric pressure mass spectrometers. On the other hand, the use of API sources, such as TPI, significantly simplifies the data analysis of this complex sample since they largely preserve the molecular or quasimolecular ion, thus avoiding complex mixed MS spectra for coeluting compounds that could hamper the correct identification. Therefore, the combination of the high separation power of GC × GC, with the soft TPI ionization and the possibility to carry out MS/HRMS experiments with a high mass accuracy, provided a powerful, high-throughput, and reliable methodology to achieve the characterization of complex samples such as VOC profile of vermouth with a high level of confidence. Using this method, the different classes of compounds were tentatively identified in the sample, including monoterpenes, terpenoids, sesquiterpenoids and carboxylic acid, and carboxylate esters as the most abundant class, followed by alkylbenzenes and phenols and aryl-aldehydes. This aroma fingerprinting might be useful not only to characterize different vermouths but also to control the quality of the products as well as to deal with potential authentication issues.

## Supplementary Information

Below is the link to the electronic supplementary material.

Supplementary file1 (DOCX 4696 KB)

## References

[1] Beckner Whitener ME, Stanstrup J, Panzeri V, Carlin S, Divol B, Du Toit M, Vrhovsek U (2016). Untangling the wine metabolome by combining untargeted SPME–GCxGC-TOF-MS and sensory analysis to profile Sauvignon blanc co-fermented with seven different yeasts. Metabolomics.

[2] Heymann H, Robinson AL, Buscema F, Stoumen ME, King ES, Hopfer H, Boulton RB, Ebeler SE. Effect of region on the volatile composition and sensory profiles of Malbec and Cabernet Sauvignon wines. In: Advances in Wine Research, vol 1203. ACS Symposium Series, vol 1203. American Chemical Society; 2015. p. 109–122. 10.1021/bk-2015-1203.ch007.

[3] Lukić I, Carlin S, Vrhovsek U (2020). Comprehensive 2D gas chromatography with TOF-MS detection confirms the matchless discriminatory power of monoterpenes and provides in-depth volatile profile information for highly efficient white wine varietal differentiation. Foods.

[4] Rocha MAM, Coimbra MA, Rocha SM, Nunes C (2021). Impact of chitosan-genipin films on volatile profile of wine along storage. Appl Sci.

[5] Weldegergis BT, Villiers Ad, McNeish C, Seethapathy S, Mostafa A, Górecki T, Crouch AM (2011). Characterisation of volatile components of Pinotage wines using comprehensive two-dimensional gas chromatography coupled to time-of-flight mass spectrometry (GC×GC–TOFMS). Food Chem.

[6] Welke JE, Zanus M, Lazzarotto M, Alcaraz Zini C (2014). Quantitative analysis of headspace volatile compounds using comprehensive two-dimensional gas chromatography and their contribution to the aroma of Chardonnay wine. Food Res Int.

[7] Bahaghighat HD, Freye CE, Synovec RE (2019). Recent advances in modulator technology for comprehensive two dimensional gas chromatography. TrAC Trends Anal Chem.

[8] Tranchida PQ, Purcaro G, Dugo P, Mondello L, Purcaro G (2011). Modulators for comprehensive two-dimensional gas chromatography. TrAC Trends Anal Chem.

[9] Krupčík J, Gorovenko R, Špánik I, Sandra P, Giardina M (2016). Comparison of the performance of forward fill/flush and reverse fill/flush flow modulation in comprehensive two-dimensional gas chromatography. J Chromatogr A.

[10] Tranchida PQ, Franchina FA, Dugo P, Mondello L (2014). Use of greatly-reduced gas flows in flow-modulated comprehensive two-dimensional gas chromatography-mass spectrometry. J Chromatogr A.

[11] Ayala-Cabrera JF, Montero L, Meckelmann SW, Uteschil F, Schmitz OJ. Review on atmospheric pressure ionization sources for gas chromatography-mass spectrometry. Part I: Current ion source developments and improvements in ionization strategies. Anal Chim Acta. 2023;1238:340353. 10.1016/j.aca.2022.340353. 10.1016/j.aca.2022.340353 36464440

[12] Ayala-Cabrera JF, Montero L, Meckelmann SW, Uteschil F, Schmitz OJ. Review on atmospheric pressure ionization sources for gas chromatography-mass spectrometry. Part II: Current applications. Anal Chim Acta. 2023;1238:340379. 10.1016/j.aca.2022.340379. 10.1016/j.aca.2022.340379 36464441

[13] Arrizabalaga-Larrañaga A, Ayala-Cabrera JF, Seró R, Santos JF, Moyano E. Ambient ionization mass spectrometry in food analysis. In: Galanakis CM, editors. Food Toxicology and Forensics. Academic Press; 2021. p. 271–312. 10.1016/B978-0-12-822360-4.00006-6.

[14] Ayala-Cabrera JF, Turkowski J, Uteschil F, Schmitz OJ (2022). Development of a tube plasma ion source for gas chromatography–mass spectrometry analysis and comparison with other atmospheric pressure ionization techniques. Anal Chem.

[15] Zhu L, Wang X, Song X, Zheng F, Li H, Chen F, Zhang Y, Zhang F. Evolution of the key odorants and aroma profiles in traditional Laowuzeng baijiu during its one-year ageing. Food Chem. 2020;310:125898 10.1016/j.foodchem.2019.125898. 10.1016/j.foodchem.2019.125898 31816535

[16] Schiewek R, Lorenz M, Giese R, Brockmann K, Benter T, Gäb S, Schmitz OJ (2008). Development of a multipurpose ion source for LC-MS and GC-API MS. Anal Bioanal Chem.

[17] Morata A, Vaquero C, Palomero F, Loira I, Bañuelos MA, Suárez-Lepe JA. Technology of vermouth wines. In: Grumezescu AM, Holban AM (eds) Alcoholic beverages. Woodhead Publishing; 2019. p. 35–63. 10.1016/B978-0-12-815269-0.00002-7.

[18] Haseeb S, Alexander B, Baranchuk A (2017). Wine and cardiovascular health. Circulation.

[19] Panighel A, Flamini R (2014). Applications of solid-phase microextraction and gas chromatography/mass spectrometry (SPME-GC/MS) in the study of grape and wine volatile compounds. Molecules.

[20] Perestrelo R, Silva C, Câmara JS. Madeira wine volatile profile. A Platform to establish madeira wine aroma descriptors. Molecules 2019;24(17):3028. 10.3390/molecules24173028 PMC6749321 31438523

[21] Marín-San Román S, Rubio-Bretón P, Pérez-Álvarez EP, Garde-Cerdán T. Advancement in analytical techniques for the extraction of grape and wine volatile compounds. Food Res Int. 2020;137:109712. 10.1016/j.foodres.2020.109712. 10.1016/j.foodres.2020.109712 33233285

[22] del Barrio Galán R, Bueno-Herrera M, de la Cuesta PL, Pérez-Magariño S. Volatile composition of Spanish red wines: effect of origin and aging time. Eur Food Res Technol. 2022;248 (7):1903-1916. 10.1007/s00217-022-04014-x.

[23] Jeleń HH, Majcher M, Dziadas M (2012). Microextraction techniques in the analysis of food flavor compounds: a review. Anal Chim Acta.

[24] Rocha SM, Costa CP, Martins C. Aroma clouds of foods: a step forward to unveil food aroma complexity using GC × GC. Front Chem. 2022;10 10.3389/fchem.2022.820749. 10.3389/fchem.2022.820749 PMC8921485 35300387

[25] Niu Y, Ma Y, Xiao Z, Zhu J, Xiong W, Chen F (2022). Characterization of the key aroma compounds of three kinds of Chinese representative black tea and elucidation of the perceptual interactions of methyl salicylate and floral odorants. Molecules.

[26] Xi J, Zhan P, Tian H, Wang P (2019). Effect of spices on the formation of VOCs in roasted mutton based on GC-MS and principal component analysis. J Food Qual.

[27] Yao C, Anderson JL (2009). Retention characteristics of organic compounds on molten salt and ionic liquid-based gas chromatography stationary phases. J Chromatogr A.

[28] Ayala-Cabrera JF, Lipok C, Li J, Moyano E, Schmitz OJ, Santos FJ. Ionic liquid stationary phase for improving comprehensive two-dimensional gas chromatographic separation of polychlorinated naphthalenes. J Chromatogr A. 2021;1635:461732 10.1016/j.chroma.2020.461732. 10.1016/j.chroma.2020.461732 33285416

[29] Davis JM, Stoll DR, Carr PW (2008). Effect of first-dimension undersampling on effective peak capacity in comprehensive two-dimensional separations. Anal Chem.

[30] Poliak M, Kochman M, Amirav A (2008). Pulsed flow modulation comprehensive two-dimensional gas chromatography. J Chromatogr A.

[31] Reiner EJ, Ladak A, Mullin L, Jobst KJ, Seeley JV (2018). Enhancing the sensitivity of atmospheric pressure ionization mass spectrometry using flow modulated gas chromatography. The Column.

[32] Schymanski EL, Jeon J, Gulde R, Fenner K, Ruff M, Singer HP, Hollender J (2014). Identifying small molecules via high resolution mass spectrometry: communicating confidence. Environ Sci Technol.

[33] Li X, Dorman FL, Helm PA, Kleywegt S, Simpson A, Simpson MJ, Jobst KJ (2021). Nontargeted screening using gas chromatography–atmospheric pressure ionization mass spectrometry: recent trends and emerging potential. Molecules.

[34] Scheubert K, Hufsky F, Böcker S (2013). Computational mass spectrometry for small molecules. J Cheminform.

[35] Ruttkies C, Schymanski EL, Wolf S, Hollender J, Neumann S (2016). MetFrag relaunched: incorporating strategies beyond in silico fragmentation. J Cheminform.

[36] FoodDB Version 1.0. www.foodb.ca. Accessed 16.03.2023.

[37] Paravisini L, Prot A, Gouttefangeas C, Moretton C, Nigay H, Dacremont C, Guichard E (2015). Characterisation of the volatile fraction of aromatic caramel using heart-cutting multidimensional gas chromatography. Food Chem.

