# Supplementary material for: Comprehensive two-dimensional gas chromatography with flow modulator coupled via tube plasma ionization to an atmospheric pressure high-resolution mass spectrometer for the analysis of vermouth volatile profile
Source: Anal Bioanal Chem. 2023 Apr 15;415(13):2561–73. doi: 10.1007/s00216-023-04688-6 (PMC10149472; doi:10.1007/s00216-023-04688-6)
Supplement: Supplementary file 1 — Supplementary file1 (DOCX 4696 KB) [file 216_2023_4688_MOESM1_ESM.docx]

Electronic supplementary material for

**Comprehensive two-dimensional gas chromatography with flow modulator coupled via tube plasma ionization to an atmospheric pressure high-resolution mass spectrometer for the analysis of vermouth volatile profile**

Juan F. Ayala-Cabrera ^1,2^, Lidia Montero ^1,2^, Taher Sahlabji ^3^, Oliver J. Schmitz ^1,2,*^

^(1)^ Applied Analytical Chemistry, University of Duisburg-Essen, Universitatsstr. 5, 45141 Essen, Germany

^(2)^ Teaching and Research Center for Separation, University of Duisburg-Essen, Universitatsstr. 5, 45141 Essen, Germany

^(3)^ Department of Chemistry, College of Science, King Khalid University, P.O. Box 9004, Abha, 61413, Saudi Arabia

**^*^**Corresponding author: Oliver J. Schmitz

**Table of Contents**

[**Supporting Tables** 2](#_Toc129877858)

[**Table S1.**  Aroma descriptors and main sources of compounds tentatively identified in vermouth. 2](#_Toc129877859)

[**Supporting Figures** 6](#_Toc129877860)

[**Figure S1.** GC×GC-HRMS chromatogram of vermouth diluted with water at a) 1:4 *v/v*, b) 1:10 *v/v*, and a) 1:100 *v/v* using a DB-5MS (30 m x 0.25 mm ID, 0.25 µm) as ^1^D column and SLB-IL60 (5 m x 0.25 mm ID, 0.20 µm) as ^2^D column. (Modulation period: 2.5 s; injection time: 0.12 s). 6](#_Toc129877861)

[**Figure S2.** Effect of the 1:100 (*v/v*) sample dilution on the low abundant compounds. Signal of the [M+H]+ ion *m/z* 153.0549 acquired by injecting the 1:4 (*v/v*) diluted sample (black) and the 1:100 (*v/v*) diluted sample (grey). 7](#_Toc129877862)

[**Figure S3.** TPI (+) mass spectrum of 2-methyl-1-phenyl-1-butanone in the vermouth sample. 7](#_Toc129877863)

# **Supporting Tables**

## **Table S1.** Aroma descriptors and main sources of compounds tentatively identified in vermouth.

| Class | Analyte | Aroma descriptor/Tasting | Source |
| --- | --- | --- | --- |
| Monoterpenes and terpenoids | Myrcene | Peppery and balsamic | Allspice, bay, hops, houttuynia, lemon grass, mango, myrcia, verbena, west Indian bay tree, and cardamom |
|  | ρ-Cymene | Woody, oxidized citrus | Cumin and thyme |
|  | D-Limonene | Citrus | Oil of citrus fruit peels, coniferous and broadleaved trees |
|  | Carveol | Minty | Spearmint, cumin, and caraway |
|  | Perillaldehyde | Cherry and fatty | Herbs and spices (cumin, caraway, ginger, lime, pepper, etc.) |
|  | Perillyl alcohol | Fruity, floral, and fatty | Essential oils of lavandin, peppermint, spearmint, cherries, celery seeds as well as caraway, ginger, camomile, and sweet bay. |
|  | Thymol | Herbal (thyme) | Essential oils (i.e., seed oil, horsemint, etc.), thyme, oregano, coriander, black walnut, and teas |
|  | Carvacrol | Oregano, spicy, and pungent | Pot and sweet marjoram, oregano, thymes, black walnuts, rosemary, pepper (spice), tamarinds, peppermints, and sweet basil |
| Sesquiterpenoids | Epicubenol | Not found | Not found |
|  | Irone | Floral, berry, violet, woody, powdery | Iris |
|  | α-Copaen-11-ol | Not found | Not found |
|  | Guaia-3,9-diene | Not found | Not found |
|  | Germacrene | Woody, spice, earth | Turmeric |
|  | Turmerone | Not found | Curcuma, turmeric |
| Carboxylic acids and carboxylate esters | Ethyl-2-methylpentanoate | Fruity apple | Fruits |
|  | 2-Propyl-2,4-pentadienoic acid | Not found | Not found |
|  | Diethyl malate | Caramel and fruity | Food additive |
|  | Methyl pentanoate | Sweet, apple, and fruity | Berries, coffee |
|  | Pentyl acetate | Banana, ethereal, and fruity | Blackberries, apples, papayas, cocoa beans |
|  | Ethyl hexanoate | Apple, pineapple, waxy, green, banana, sweet | Wine, apple, pear, pomes, passion fruit, cherry, citrus |

**Table S1 (cont.).** Aroma descriptors and main sources of compounds tentatively identified in vermouth.

| Carboxylic acids and carboxylate esters | Ethyl furoate | Plum | Not found |
| --- | --- | --- | --- |
|  | Ethyl levulinate | Berry, floral, sweet | Blackberries |
|  | Ethyl sorbate | Fruity, anise, licorice, green, tropical, pineapple | Tropical fruits |
|  | Octyl acetate | Citrus, fruity | Citrus peel, wines, banana, sour cherry |
|  | Methyl nonanoate | Pear, tropical, fruity | Apples, bananas, blackberries, grapes, pineapples, strawberries, vanilla |
|  | Ethyl phenylacetate | Sweet, anise, balsam | Citrus, chamomile |
|  | Diethyl glutarate | Not found | Not found |
|  | Octyl propanate | Not found | Not found |
|  | Methyl decanoate | Wine, fruity, floral, oily | Pome, tropical fruit, berries |
|  | Cys-Chrysanthenin propionate | Not found | Artemisia |
|  | Hexyl hexanoate | Appel, peach, herbal, fruity | Apple, pear, pomes, tropical fruit, cherry, citrus |
|  | Isobutyl 2-furanpropionate | Fruity, floral, woody, pineapple, sweet | Not found |
|  | Methyl dodecanoate | Floral, sweet | Grape, melon, pineapple, blackberry |
|  | Isoamyl salicylate | Bitter, floral, herbal | Tea, fruits |
| Aryl-aldehydes | Furfural | Sweet, almond. bready | Product of dehydration of sugars, Poaceae, coffee, Theobroma cacao |
|  | 5-methylfurfural | Almond, burnt sugar and caramel | Red raspberries, pepper, evergreen blackberries |
|  | Benzaldehyde | Almond | Black walnuts, almonds, corns, safflowers, kohlrabis, Ceylon cinnamons |
|  |  |  |  |
|  | Heptadienal | Cake, cinnamon | Blackberries |
|  | Hydroxy-anisaldehyde | Not found | Not found |
|  | Piperonal | Cherry, vanilla, almond, tropical, bitter, spicy | Berries, melon, pepper, vanilla |
| Alkylbenzenes and phenols | Cis-1,2-dihydro-3-ethylcatechol | Not found | Not found |
|  | o-/m-/ρ-cresol | Fecal, leather, and medicinal | Teas (*camellia sinensis*), green tee, arabica coffees |

**Table S1 (cont.).** Aroma descriptors and main sources of compounds tentatively identified in vermouth.

| Alkylbenzenes and phenols | Chavicol | Medicinal and phenolic | Cloves, sweer marjoram, sweet basils, pineapples, allspices, Chinese cinnamons, and gingers |
| --- | --- | --- | --- |
|  | Styrene | Sweet, balsamic, and floral | Coffee, fruits, cocoa, alcoholic beverages, and Chinese cinnamons |
|  | Cymene | Citrus | Citrus, cumin, thyme essential oils |
|  | 3-Methylene-1,5,5- trimethylcyclohexene | Not found | Not found |
|  | Eugenyl formate | Warm, woody | Flavoring agent |
|  | Eugenol | Spicy, clove-like, and pungent | Cloves, allspices, carrots, walnuts, Ceylon cinnamons, nutmeg, basil, and bay leaf |
|  | 3-Methyl-1-phenyl-3-pentanol | Sweet and anise | Not Found |
|  | 1,3,5,5-Tetramethyl-1,3-cyclohexadiene | Not found | Not found |
|  | Methyl eugenol | Anise, apricot, chocolate, vanilla, sweet | Pepper, cloves, cinnamon, rosemary, cherry, citrus, blackberry |
|  | Vanillin | Vanilla, chocolate, creamy, sweet, | Cinnamon, ginger, pepper, cloves, vanilla, nuts, apple, pear, peach, cherry, banana, tropical fruits, berries, citrus |
| Alcohols and alcohols esters | Trans-lachnophyllol | Not Found | Plant (*Aster tataricus*) |
|  | Citronellyl acetate | Floral (rose) and citrus | Not Found |
|  | Cinnamyl alcohol | Sweet, balsam, bitter | Cinnamon, anis |
|  | Ginsenol | Not found | Gingsen |
| Heterocyclic compounds | Fructone | Fruity (Apple, pineapple, strawberry) and woody | Flavourant |
|  | Nonalactone | Fruity | Fruits (peaches and apricots), and Bourbon whiskey |
|  | Piperonal | Floral (vanillin or cherry) | Plants (dill, vanilla, violet flower, and black pepper), blueberries, and pepper (spice) |
|  | 3-(3-Furanyl)-2-methyl-2-propenal | Not found | Not found |

**Table S1 (cont.).** Aroma descriptors and main sources of compounds tentatively identified in vermouth.

| Ketones | Dihydrocarvanone | Minty, green | Dill, mentha, spearmint |
| --- | --- | --- | --- |
|  | 2-Methyl-1-phenyl-1-butanone | Not Found | Not Found |
| Benzoic acids | Methyl benzoate | Floral, violet |  |
|  | Ethyl benzoate | Sweet, anise, balsam | Elderberries, pomes, allspices, blackcurrants |
| Benzopyranes | Edulan I | Not found | Fruits |

# **Supporting Figures**

## **Figure S1.** GC×GC-HRMS chromatogram of vermouth diluted with water at a) 1:4 *v/v*, b) 1:10 *v/v*, and a) 1:100 *v/v* using a DB-5MS (30 m x 0.25 mm ID, 0.25 µm) as ^1^D column and SLB-IL60 (5 m x 0.25 mm ID, 0.20 µm) as ^2^D column. (Modulation period: 2.5 s; injection time: 0.12 s).

## **Figure S2.** Effect of the 1:100 (*v/v*) sample dilution on the low abundant compounds. Signal of the [M+H]+ ion *m/z* 153.0549 acquired by injecting the 1:4 (*v/v*) diluted sample (black) and the 1:100 (*v/v*) diluted sample (grey).

## **Figure S3.** TPI (+) mass spectrum of 2-methyl-1-phenyl-1-butanone in the vermouth sample.
